# Want of Sleep

**Published:** 1871-06

**Authors:** 


### WANT OF SLEEP.

A NEW death-dealing drug has been brought to the public attention, called the "Chloral Hydrate." It is a valuable medicine when especially advised by an experienced physician, but when taken on the responsibility of any one who may want to sleep a little better, it is uncertain, if taken in a liquid form. If it has been long in a fluid state, it begins to deteriorate from the very first hour of its solution ; while a great many liquids deprive it of its peculiar power. When taken in its pure state it has an admirable effect, inducing sleep ; but if taken often, taken daily, it in a short time becomes poisonous, inducing very serious and critical results. As it acts on the brain, mainly, perhaps the unprofessional should avoid its use. It is a dangerous experiment for any one in ordinary health to get into the habit of inducing sleep by any other means than moderate exercise or work.

